# The Effect of Al_2_O_3_ Nanoparticles on Hexagonal Boron Nitride Films Resulting from High-Temperature Annealing

**DOI:** 10.3390/nano15070484

**Published:** 2025-03-24

**Authors:** Qiang Li, Kangkang Liu, Ransheng Chen, Wannian Fang, Zhihao Zhang, Youwei Chen, Haifeng Liu, Ziyan Lin, Yuhuai Liu, Tao Wang

**Affiliations:** 1 Key Laboratory of Physical Electronics and Devices for Ministry of Education and Shaanxi Provincial Key Laboratory of Photonics & Information Technology, Xi’an Jiaotong University, Xi’an 710049, China; liukang4142@stu.xjtu.edu.cn; 2 School of Electronic Science and Engineering, Xi’an Jiaotong University, Xi’an 710049, China; chenransheng@stu.xjtu.edu.cn (R.C.); wannian333@stu.xjtu.edu.cn (W.F.); zzh1920676733@stu.xjtu.edu.cn (Z.Z.); 202194611912@stu.xjtu.edu.cn (Y.C.); scorpio@stu.xjtu.edu.cn (H.L.); lzy2213525329@stu.xjtu.edu.cn (Z.L.); 3 National Centre for International Joint Research of Electronic Materials and Systems, School of Information Engineering, Zhengzhou University, Zhengzhou 450001, China; 4 School of Engineering, Nagoya University, Nagoya 464-8601, Aichi, Japan; 5 School of Physics and Astronomy, Cardiff University, Cardiff CF24 3AA, UK

**Keywords:** boron nitride, magnetron sputtering, post-annealing

## Abstract

A simple two-step approach was proposed to obtain hBN thin films with high crystalline quality, meaning that the films were initially prepared by using an RF magnetron sputtering technique and subsequently followed by a post-annealing process at a high temperature. In the case of introducing Al_2_O_3_ nanoparticles, the effects of annealing temperature from 1000 °C to 1300 °C and annealing time from 0.5 h to 1.5 h on the recrystallization process of the grown hBN films were systematically studied by using XRD and SEM technologies. The introduction of Al_2_O_3_ impurities during the annealing process successfully reduced the transition temperature of hexagonal phase BN by more than 300 °C. The crystalline quality of hBN films grown by RF magnetron sputtering could be effectively enhanced under annealing at 1100 °C for 1 h. The DUV detectors were prepared using the hBN films before and after annealing, and showed a notable improvement in detector performance by using annealed films. It has significant application value in further enhancing the performance of DUV photodetectors based on high-quality hBN films.

## 1. Introduction

Hexagonal boron nitride (hBN), as a synthetic ultra wide-band semiconductor material, exhibits excellent electrical, optical and mechanical properties [1,2,3,4,5,6]. The band gap up to 6 eV presents great potential in luminescence and detection in the deep-ultraviolet spectral region [7,8,9]. The extremely high dielectric strength (~8 MV/cm) makes it an ideal dielectric candidate for the fabrication of switching and memory devices [10,11,12]. The high thermal conductivity, antioxidant properties, and chemical stability endow hBN with the ability of being used as a thermally conductive and heat-resistant material, coating protection material and a variety of functional composite materials [13,14]. The two-dimensional graphene-like layered structure associated with van der Waals forces of hBN offers an advantage in the fabrication of innovative electronic devices such as flexible electronic devices [12,13,14,15].

A number of approaches have been proposed to synthesize hBN. In general, there are mainly two methods for the fabrication of hBN films. The first one is to obtain nanosheet films via mechanical stripping or liquid phase stripping from hBN bulk materials [16,17,18,19], which exhibits the physical and chemical properties approaching the theoretical value of single-crystal hBN. However, it is difficult to control the size, shape and thickness of nanosheets, and thus they are impractical to be used for mass production purposes. The second approach is to use an epitaxial technique on a substrate via certain precursors and reaction processes, such as chemical vapor deposition (CVD), molecular beam epitaxy (MBE), sputtering deposition, etc. [20,21,22,23,24,25,26,27]. The major advantage of these methods is related to their capability of growing a continuous hBN film on a large scale. However, the epitaxy growth generally poses challenges such as lattice mismatch and thermal mismatch between hBN and a foreign substrate used, inevitably affecting the crystallinity of the hBN films grown on its top. Among these methods, radio frequency (RF) magnetron sputtering demonstrates major advantages from the perspective of the rapid growth of thick films on a large scale at a low temperature (600–800 °C). Unfortunately, it should be noted that the crystalline quality of hBN films is greatly limited by the relatively low growth temperature (600–800 °C) [28]. It is generally expected that high-temperature annealing can potentially enhance the crystal quality of semiconductors [29,30,31,32], which has also been proven to be valid in preparing high-quality hBN thin films [24,33,34,35,36,37]. However, integrating a high-temperature annealing process into RF magnetron sputtering still remains a great challenge, and thus needs to be investigated.

Generally speaking, it is requested that either the growth of hBN thin films with reasonable or post-annealing is conducted at a temperature of more than 1400 °C, posing technical challenges. Therefore, it is necessary to develop a new approach to reduce the required temperature, which has been the main focus for researchers [33,34,35,36,37].

Herein, a two-step method consisting of initial growth at a low temperature using RF magnetron sputtering and a subsequent high-temperature annealing process is proposed, aiming to synthesize high-quality, large-area, continuous hBN films. This method has achieved great success in other semiconductor systems, such as AlN films grown on sapphire [38,39]. Moreover, in this paper, we also introduce Al_2_O_3_ nano particles during the annealing process, leading to not only a reduction in annealing temperature (down to 1100 °C) but also an improvement in crystalline quality. Radial morphology was observed on the surface of the annealed samples, and the formation mechanism is discussed. Finally, deep-ultraviolet (DUV) detectors were fabricated by using hBN films before and after annealing. The better photoelectric performance with annealed hBN film verified the improvement of hBN film crystal quality.

## 2. Materials and Methods

The growth of hBN films was triggered by means of a RF magnetron sputtering technique. High-purity nitrogen (99.9999%) as a supplementary nitrogen source was introduced to make up for the nitrogen deficiency problem that exists in the sputtering process [40], and argon was used as a glow gas. At least three rounds of gas flush processes were initially carried out to ensure that the air in the furnace was entirely removed to prevent any oxidation. The hBN target with 99.9% purity and double-sided polished sapphire substrate (c-plane) were used. After heating the substrate to 600 °C, a sputtering process was conducted at 250 W for 8000 s. During the sputtering process, the pressure was controlled at 0.6 Pa, where the flow rates of argon and nitrogen were 30 sccm and 10 sccm, respectively. The thin films with a thickness of 187 nm were obtained, showing a refractive index of 1.72.

During the annealing process, high-purity nitrogen was introduced at a gas flow rate of 50 sccm and the chamber pressure was maintained at 1 torr. Two pieces of samples were placed together in a face-to-face configuration to effectively reduce decomposition and volatilization at high temperatures, ensuring film integrity after annealing [39,41]. Aluminum oxide (Al_2_O_3_) particles, especially α- Al_2_O_3_, have excellent stability at high temperatures [42]. Therefore, Al_2_O_3_ particles were innovatively introduced as oxide impurities to reduce the phase transition temperature, based on the previous study of the influence of impurities on the phase transition temperature of boron nitride [43,44,45,46,47]. Ellipsometry (EMpro from Ellitop, Beijing, China), X-Ray Diffraction (XRD, Bruker D8 Advance, Bruker Scientific Instruments, Ettlingen, Germany) and Scanning Electron Microscope (SEM, Gemini SEM 500, Carl Zeiss Shanghai Management Co., Ltd., Shanghai, China) have been used to characterize the thickness, crystalline quality, and surface morphology of both as-grown and annealed hBN films, respectively.

## 3. Results and Discussion

**Figure 1:**
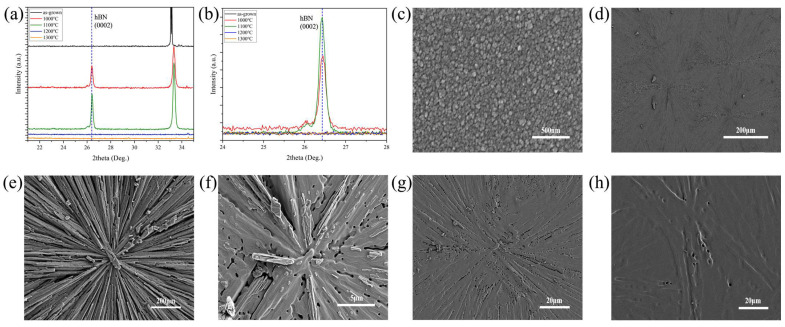
(**a**) XRD characterization results of thin films before and after annealing at different temperatures for 1 h; (**b**) partial enlargement. SEM morphology characterization of the sample’s surface before and after annealing at different temperatures for 1 h: (**c**) pristine sample; (**d**) overall surface morphology after annealing at 1000 °C; (**e**) sample annealed at 1000 °C; (**f**) sample annealed at 1100 °C; (**g**) sample annealed at 1200 °C; (**h**) sample annealed at 1300 °C.

In order to clarify the effect of annealing parameters on the film crystal quality following the introduction of Al_2_O_3_ nanoparticles, different annealing temperatures (1000–1300 °C) and times (0.5–1.5 h) were selected for the study. Figure 1a,b display the XRD characterization results of the films obtained before and after annealing, indicating a diffraction peak at 26.4° for the sample annealed at 1000 °C or 1100 °C corresponding to the diffraction peak of hBN (0002). In contrast, such a peak did not appear on the pristine sample and the samples annealed at the other higher temperatures. These results indicated that high crystalline quality can be achieved by the annealing treatment even at 1000 °C or 1100 °C. Moreover, the characteristic peak of (0002) for the sample annealed at 1100 °C exhibited an approximately 59.6% higher intensity than that of the sample annealed at 1000 °C. The XRD spectrum of the sample annealed at 1100 °C shows a full width at half maximum (FWHM) of 0.16°, which is narrower than that of the sample annealed at 1000 °C (0.18°). All this meant that the optimized annealing temperature is 1100 °C.

Figure 1c–h present the SEM images of these samples. It can be seen from Figure 1c that the as-grown film features many nano-particles, which is consistent with the island growth model of hBN films [28]. Figure 1d shows many radial patterns after the sample underwent annealing at 1000 °C for 1 h, while Figure 1e provides a high-definition image for the local part of the sample. Figure 1f–h present the morphology evolution when the annealing temperatures was increased from 1100 °C to 1200 °C and 1300 °C.

**Figure 2:**
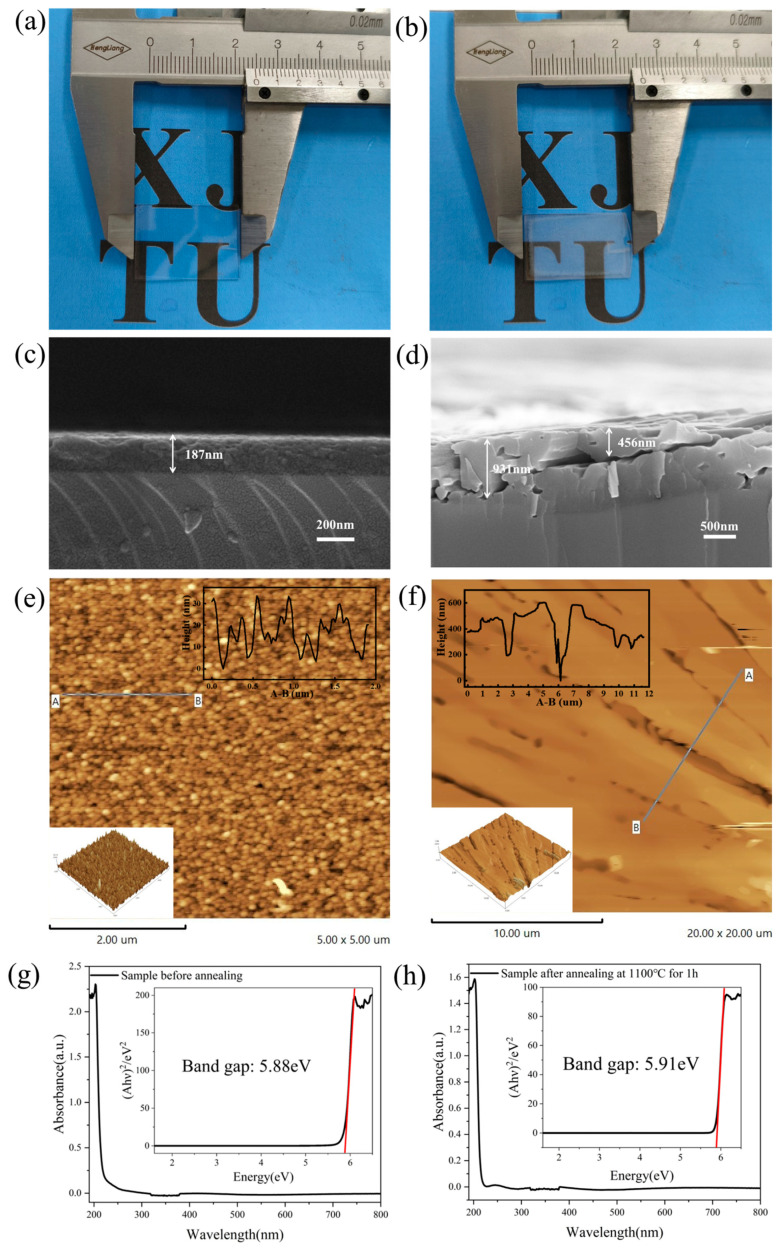
Some characteristics of the samples before and after annealing at 1100 °C for 1 h: (**a**,**b**) photographs of samples; (**c**,**d**) cross-sectional SEM of the samples; (**e**,**f**) AFM characterization of samples; The letters (A,B) are the two ends of a line segment chosen to represent the roughness of films. Their actual physical positions are shown by the straight line in the figure. The specific thickness changes are displayed in the inset at the top-left corner. The x-axis in the inset corresponds to A-B. (**g**,**h**) absorption spectra and bandgap of samples.

For detailed comparison, the characteristics of the samples before and after annealing are provided in Figure 2. Figure 2a,b show the photographs of samples, indicating that the films demonstrated a situation similar to “hairy glass” after annealing. Figure 2c shows the cross section of hBN pristine film with a thickness of 187 nm. Figure 2d indicates that the film became thicker after annealing, due to pore formation and film loosening during recrystallization. AFM measurement was used to validate the radial morphological changes, and the results are shown in Figure 2e,f. Figure 2e shows the pristine sample with the root mean square (RMS) error of 7.91 nm, indicating an extremely smooth surface from sputtered film growth. The post-annealing sample with a rough surface (RMS of 110.11 nm in the gully region) is shown in Figure 2f. Surface morphological changes from AFM results were consistent with the conclusion of the SEM measurement. Meanwhile, absorption spectra tests before and after annealing at the optimal parameters were performed. The results are shown in Figure 2g,h and the insets represent the optical bandgap, which indicates that the annealing process had little effect on the intrinsic absorption peak of the films.

**Figure 3:**
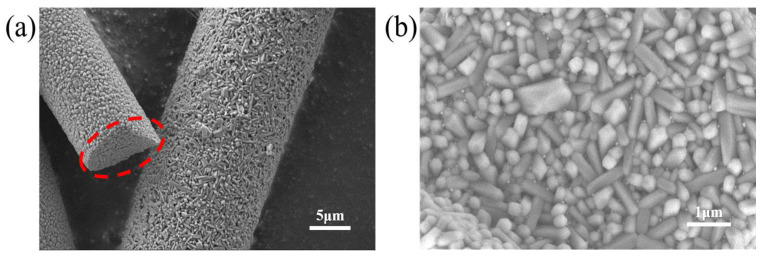
SEM morphology characterization of (**a**) corundum powders and (**b**) Al_2_O_3_ nanoparticles. The circle in the figure indicates that Figure 3b is the enlarged view of the circled area.

This phenomenon was attributed to the introduction of Al_2_O_3_ particles. The inner wall of the annealing furnace was coated with a layer of micrometer-sized rod-shaped Al_2_O_3_ powders. The SEM morphology characterization of the corundum powders is shown in Figure 3a, and the surface of the powders was covered with Al_2_O_3_ nanoparticles, as shown in Figure 3b. At high temperatures, Al_2_O_3_ nanoparticles formed on the surface of microrod powders and fell off due to gas-flow disturbance. Although the samples were placed in a face-to-face manner, the Al_2_O_3_ nanoparticles could also be absorbed by the hBN film with the continuous flow of nitrogen gas. These impurities acted as condensation nuclei, inducing a high-temperature recrystallization process of hBN films that encompassed this core and thus eventually forming radial patterns. With the temperature rising further, the radial pattern on the surface began to melt, causing the surface of the film to be flat again. It should be noted that a higher temperature (such as 1200 °C and 1300 °C) weakens the influence of the Al_2_O_3_ nanoparticles during the transition of the film to the hexagonal phase, while the characteristic peaks in the XRD disappeared if the temperature did not reach a certain level (>1400 °C) that caused a spontaneous transition to the hexagonal phase.

**Figure 4:**
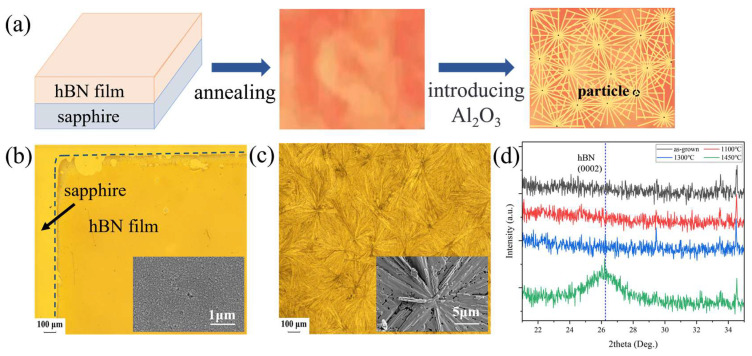
(**a**) Schematic diagram of the generation of radial patterns on the surface of the film after annealing. Characterization of thin films after annealing in the tubular high-temperature furnace with a light microscope on the sample surface: (**b**) sample annealed at 1450 °C for 1 h without Al_2_O_3_ nanoparticles; (**c**) sample annealed at 1100 °C for 1 h with Al_2_O_3_ nanoparticles; (**d**) XRD characterization results of thin films before and after annealing at different temperatures for 1 h in the high-temperature tube furnace.

**Figure A1:**
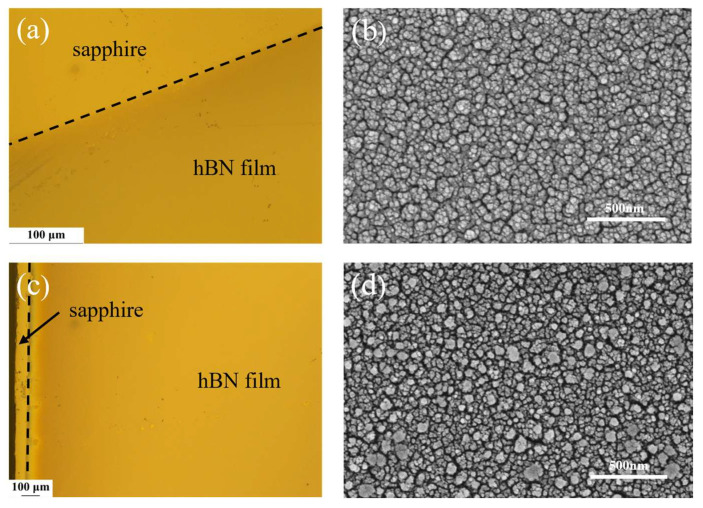
Light microscope and SEM of the sample surface: (**a**,**b**) sample annealed at 1100 °C for 1h in the tubular high-temperature furnace without Al_2_O_3_ nanoparticles; (**c**,**d**) sample annealed at 1300 °C for 1 h in the tubular high-temperature furnace without Al_2_O_3_ nanoparticles.

Figure 4a illustrates the whole process schematically. To further demonstrate the role of Al_2_O_3_ nanoparticles, a high-temperature tube furnace without a microrod corundum powder coating was used for annealing. The temperatures were set to 1100 °C, 1300 °C, and 1450 °C with the same annealing time and atmosphere. The surface morphology of the sample annealing at 1450 °C is shown in Figure 4b and the samples annealed at different temperatures exhibited similar morphologies, which are shown in Figure A1. For comparison, the sample annealed in the previously described annealing furnace is displayed in Figure 4c. The surfaces of the films did not show any radial patterns. A borderline between the hBN film and the sapphire substrate was clear to be observed, indicating that the film maintained a continuous and smooth surface even at a higher temperature. The crystalline quality was also characterized by XRD, as shown in Figure 4d. The characteristic peaks of hBN (0002) appeared only for the sample annealed at 1450 °C, but the samples annealed at 1100 °C and 1300 °C did not show this peak. Hence, these results confirmed that the aforementioned Al_2_O_3_ particles work as condensation nuclei to induce recrystallization and improve the crystallinity of the hBN film. Although the introduction of Al_2_O_3_ nanoparticles caused the radial morphology, it had effectively led to a reduction in annealing temperature by more than 300 °C.

**Figure 5:**
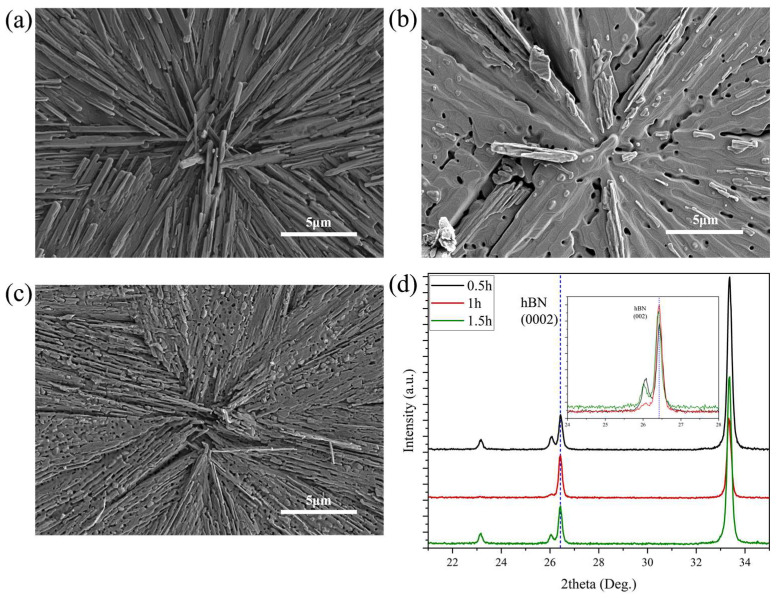
SEM morphology characterization of film surface annealed at 1100 °C at different times: (**a**) 0.5 h; (**b**) 1 h; (**c**) 1.5 h. (**d**) XRD characterization of thin films annealed at 1100 °C for different time and its partial enlargement.

The effect of annealing time on the quality of thin film crystals was also investigated. Annealing time was chosen to be 0.5 h, 1 h and 1.5 h at 1100 °C while the other experimental conditions remained unchanged. The surface morphology of these samples is shown in Figure 5a–c. It can be found that the radial patterns always appeared regardless of annealing time. The XRD results of the samples annealed for different times are shown in Figure 5d. The characteristic peak of hBN (0002) appeared at 26.4° for all the annealed samples. The sample annealed for 1 h showed the highest intensity of the characteristic peak compared to the other two types of samples. The peak intensity was approximately 20.7% higher than that of the sample annealed for 0.5 h, and 11.8% higher than that of the sample annealed for 1.5 h. The FWHM of the XRD peaks of these annealed samples was 0.18°, 0.16° and 0.18° for an annealing time of 0.5 h, 1 h and 1.5 h, respectively. The sample annealed for 0.5 h showed insufficient recrystallization as a result of the short time, while the sample annealed for 1.5 h exhibited the decomposition of the film due to the excessive annealing time. Notably, a shoulder peak was observed at the left of the hBN (0002) characteristic peak for all the annealed samples, which possibly corresponds to the (012) characteristic peak of the introduced Al_2_O_3_ nanoparticles. To sum up, with the introduction of Al_2_O_3_ nanoparticles, setting the annealing conditions to 1100 °C and 1 h effectively leads to the higher crystallization quality of hBN films.

**Figure 6:**
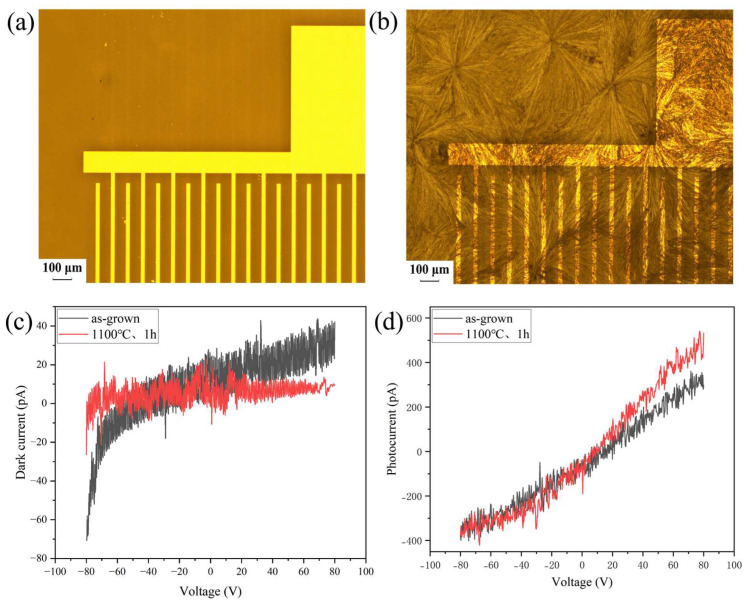
Preparation of finger electrodes by photolithography before and after annealing: (**a**) unannealed film; (**b**) annealed film at 1100 °C for 1 h. Characterization of (**c**) dark current and (**d**) photocurrent for different samples at 185 nm UV irradiation.

In order to further validate the effect of crystalline quality on the performance of devices, the hBN films annealed under optimal conditions (1100 °C for 1 h) were used to fabricate DUV photodetectors. For comparison, un-annealed samples were fabricated into devices using the same process. Nickel (Ni) metal finger electrodes were made with the samples by photolithography, and their UV detection performances were tested. Figure 6a,b shows the optical micrograph of the finger electrodes from both unannealed and annealed samples, and the light current response was measured under 185 nm UV irradiation. The response characteristics are shown in Figure 6c,d. The dark current of the annealed sample was lower and stable with increasing voltage. The photocurrent was slightly better than that of the unannealed sample at a positive voltage. Under a bias voltage of 80 V, the dark currents of the photodetectors were measured at 32.5 pA and 9.3 pA and the photo currents were recorded as 297 pA and 532 pA, respectively. Consequently, the photo-to-dark current ratios were 9.1 (for the unannealed sample) and 57.2 (for the annealed sample), respectively. Meanwhile, the responsivities of the DUV photodetectors made from the samples before and after annealing were calculated to be 0.38 mA/W and 0.68 mA/W under a bias voltage of 80 V, respectively. It is undeniable that the surface of the annealed sample tends to be rough, potentially leading to a degradation in electrode contact quality. This affected electrical performance. However, the performance of the device fabricated from the annealed thin film was still higher than that of the device fabricated from the unannealed sample. This further confirmed that an improvement in crystal quality of thin films can be achieved due to annealing, eventually leading to a great enhancement in device characteristics.

## 4. Conclusions

In summary, a two-step method was employed to prepare hBN thin films with high crystalline quality, including the initial growth using an RF magnetron sputtering technique and then high-temperature post-annealing. During the annealing processes, Al_2_O_3_ nanoparticles were introduced, where the effects of annealing temperature and annealing time on the recrystallization process of the grown hBN films were systematically studied. The introduction of Al_2_O_3_ nanoparticles during the annealing process successfully reduced the transition temperature of hexagonal phase BN. The crystalline quality of the hBN films grown by RF magnetron sputtering could be effectively enhanced after they underwent the annealing process at 1100 °C for 1 h. DUV detectors were fabricated using the hBN films with and without the annealing processes, demonstrating a significant improvement in device performance for the devices fabricated from the annealed film. It has potential application value in further enhancing the performance of DUV photodetectors based on high-quality hBN films.

## Data Availability

The raw data supporting the conclusions of this article will be made available by the authors on request.

## Appendix A

The morphologies of samples annealed at 1100 °C and 1300 °C for 1h in the tubular high-temperature furnace without Al_2_O_3_ nanoparticles.

## References

[1] Jiang X.F., Weng Q., Wang X.B., Li X., Zhang J., Golberg D., Bando Y. (2015). Recent Progress on Fabrications and Applications of Boron Nitride Nanomaterials: A Review. J. Mater. Sci. Technol..

[2] Roy S., Zhang X., Puthirath A.B., Meiyazhagan A., Bhattacharyya S., Rahman M.M., Babu G., Susarla S., Saju S.K., Tran M.K. (2021). Structure, Properties and Applications of Two-Dimensional Hexagonal Boron Nitride. Adv. Mater..

[3] Liu L., Feng Y., Shen Z. (2003). Structural and Electronic Properties of h-BN. Phys. Rev. B.

[4] Satawara A.M., Shaikh G.A., Gajjar P.N., Gupta S.K. (2020). Structural, Electronic and Optical Properties of Hexagonal Boron-nitride (h-BN) Monolayer: An Ab-initio study. Mater. Today Proc..

[5] Zhang H., Chen Y., Ma J., Tong H., Yang J., Ni D., Hu H., Zheng F. (2011). A Simple Thermal Decomposition-nitridation Route to Nanocrystalline Boron Nitride (BN) from a Single N and B Source Precursor. J. Alloys Compd..

[6] Kobayashi Y., Tsai C.-L., Akasaka T. (2010). Optical Band Gap of h-BN Epitaxial Film Grown on c-plane Sapphire Substrate. Phys. Status Solidi C.

[7] Watanabe K., Taniguchi T., Kanda H. (2004). Direct-bandgap Properties and Evidence for Ultraviolet Lasing of Hexagonal Boron Nitride Single Crystal. Nat. Mater..

[8] Fang W., Li Q., Zhang Q., Chen R., Li J., Liu K., Yun F. (2024). Vacuum Ultraviolet Photodetectors with MSM Structure Based on Hexagonal Boron Nitride Films via Magnetron Sputtering (Invited). Acta Photon. Sin..

[9] Zhang Q., Li Q., Chen R., Zhang M., Fang W., Li J., Wang M., Yun F., Wang T., Hao Y. (2024). Large-Area Self-Assembled Hexagonal Boron Nitride Nanosheet Films for Ultralow Dark Current Vacuum-Ultraviolet Photodetectors. Adv. Funct. Mater..

[10] Jiang Y., Huang Y., Zhang S. (2022). Synthesis of Hexagonal Boron Nitride Thin Film on Pt Substrates for Resistive Switching Memory Applications. Curr. Appl. Phys..

[11] Kim M., Pallecchi E., Ge R., Wu X., Ducournau G., Lee J.C., Happy H., Akinwande D. (2020). Analogue Switches Made from Boron Nitride Monolayers for Application in 5G and Terahertz Communication Systems. Nat. Electron..

[12] Guo N., Wei J., Jia Y., Sun H., Wang Y., Zhao K., Shi X., Zhang L., Li X., Cao A. (2013). Fabrication of Large Area Hexagonal Boron Nitride Thin Films for Bendable Capacitors. Nano Res..

[13] Wu W., Liu J., Liu J., Zou Z., Zhang X. (2019). Preparation and Properties of Thermally Conductive Co-POM Materials Filled with Composite of h-BN and Al_2_O_3_. J. East China Univ. Sci. Technol..

[14] Chen X., Wu Y., Wu Z., Han Y., Xu S., Ye W., Han T., He Y., Cai Y., Wang N. (2015). High-quality Sandwiched Black Phosphorus Heterostructure and its Quantum Oscillations. Nat. Commun..

[15] Zhang K., Feng Y., Wang F., Yang Z., Wang J. (2017). Two-Dimensional Hexagonal Boron Nitride (2D-hBN): Synthesis, Properties and Applications. J. Mater. Chem. C.

[16] Novoselov K.S., Jiang D., Schedin F., Booth T.J., Khotkevich V.V., Morozov S.V., Geim A.K., Rice T.M. (2005). Two-Dimensional Atomic Crystals. Proc. Natl. Acad. Sci. USA.

[17] Gorbachev R.V., Riaz I., Nair R.R., Jalil R., Britnell L., Belle B.D., Hill E.W., Novoselov K.S., Watanabe K., Taniguchi T. (2011). Hunting for Monolayer Boron Nitride: Optical and Raman Signatures. Small.

[18] Wang N., Yang G., Wang H., Yan C., Sun R., Wong C.-P. (2019). A Universal Method for Large-Yield and High-Concentration Exfoliation of Two-Dimensional Hexagonal Boron Nitride Nanosheets. Mater. Today.

[19] Zhang C., Tan J., Pan Y., Cai X., Zou X., Cheng H.-M., Liu B. (2020). Mass Production of 2D Materials by Intermediate-assisted Grinding Exfoliation. Natl. Sci. Rev..

[20] Quan H., Wang X., Zhang L., Liu N., Feng S., Chen Z., Hou L., Wang Q., Liu X., Zhao J. (2017). Stability to Moisture of Hexagonal Boron Nitride Films Deposited on Silicon by RF Magnetron Sputtering. Thin Solid Films.

[21] Chen L., Tai J., Wang D., Wang S., Liang H., Yin H. (2024). High-performance Solar-blind Photodetector Based on Amorphous BN in Harsh Environment Operations. Appl. Phys. Lett..

[22] Wu C., Soomro A.M., Sun F., Wang H., Huang Y., Gao N., Chen X., Kang J., Cai D., Wu J. (2016). Large-roll Growth of 25-inch Hexagonal BN Monolayer Film for Self-release Buffer Layer of Free-standing GaN Wafer. Sci. Rep..

[23] Yang X., Nitta S., Nagamatsu K., Bae S.-Y., Lee H.-J., Liu Y., Pristovsek M., Honda Y., Amano H. (2018). Growth of Hexagonal Boron Nitride on Sapphire Substrate by Pulsed-Mode Metalorganic Vapor Phase Epitaxy. J. Cryst. Growth.

[24] Liu F., Rong X., Sheng B.W., Wei J.Q., Liu S.F., Yang J.J., Xu F.J., Yang X.L., Zhang Z.H., Qin Z.X. (2020). Thermally Annealed Wafer-scale h-BN Films Grown on Sapphire Substrate by Molecular Beam Epitaxy. Appl. Phys. Lett..

[25] Li Y., Lin Z., Zheng W., Huang F. (2021). Micron-Thick Hexagonal Boron Nitride Crystalline Film for Vacuum Ultraviolet Photodetection with Improved Sensitivity and Spectral Response. ACS Appl. Electron. Mater..

[26] Sun X., Feng Y., Wang F., Wang P., Gao W., Yin H. (2022). Direct Growth of h-BN Multilayers with Controlled Thickness on Non-Crystalline Dielectric Substrates Without Metal Catalysts. Chem. Commun..

[27] Wang S., Liu X., Yu H., Liu X., Zhao J., Hou L., Gao Y., Chen Z. (2024). Transfer-Free Analog and Digital Flexible Memristors Based on Boron Nitride Films. Nanomaterials.

[28] Thornton J.A. (1975). Influence of Substrate Temperature and Deposition Rate on Structure of Thick Sputtered Cu Coatings. J. Vac. Sci. Technol..

[29] Gansukh M., Martinho F., Espindola M., Engberg S., Schou J., Canulescu S. (2024). The Effect of Post-Annealing on the Performance of the Cu_2_ZnSnS_4_ Solar Cells. Sci. Rep..

[30] Xiao R., Cheng J., Lu Z., Sun Q., Wang X., Fu X., Gao J. (2024). Impact of In-doping and Post-Annealing on the Properties of SnO_2_ Thin Films Deposited by Magnetron Sputtering. Phys. Scr..

[31] Liu Z., Wang R.X. (2024). Effect of Post-annealing Treatment on Structural, Optical and Photocatalytic Properties of TiO_2_ Nanoparticles Prepared via Pulsed Laser Ablation in Liquid. J. Ovonic Res..

[32] Wei C., Liu J., Lan X., Yang C., Huang S., Wang X., Chen D. (2024). The Fabrication of Ultra-Wide Bandgap GeO_2_ Thin Films by DC Magnetron Sputtering: The Impacts of Growth Temperature and Post-Annealing Process. Vacuum.

[33] Liu F., Yu J., Bai X. (2012). Crystallinity Improvement of Hexagonal Boron Nitride Films by Molybdenum Catalysts During Microwave Plasma Chemical Vapor Deposition and Post-Annealing. Appl. Surf. Sci..

[34] Lee S.H., Jeong H., Okello O.F.N., Xiao S., Moon S., Kim D.Y., Kim G.-Y., Lo J.-I., Peng Y.-C., Cheng B.-M. (2019). Improvements in Structural and Optical Properties of Wafer-Scale Hexagonal Boron Nitride Film by Post-Growth Annealing. Sci. Rep..

[35] Deng J.-X., Zhang X.-K., Yao Q., Wang X.-Y., Chen G.-H., He D.-Y. (2009). Optical Properties of Hexagonal Boron Nitride Thin Films Deposited by Radio Frequency Bias Magnetron Sputtering. Chin. Phys. B.

[36] Valerius P., Herbig C., Will M., Michely T., Arman M.A., Knudsen J., Caciuc V., Atodiresei N. (2017). Annealing of Ion-irradiated Hexagonal Boron Nitride on Ir(111). Phys. Rev. B.

[37] Singhal R., Echeverria E., McIlroy D.N., Singh R.N. (2022). Post-Growth Enhancement of CVD-Grown Hexagonal Boron Nitride Films on Sapphire. Res. Mater..

[38] Solonenko D., Schmidt C., Zahn D.R.T., Stoeckel C., Hiller K. (2020). The Limits of the Post-Growth Optimization of AlN Thin Films Grown on Si(111) via Magnetron Sputtering. Phys. Status Solidi B Basic Res..

[39] Zaiter A., Michon A., Nemoz M., Courville A., Vennéguès P., Ottapilakkal V., Vuong P., Sundaram S., Ougazzaden A., Brault J. (2022). Crystalline Quality and Surface Morphology Improvement of Face-to-Face Annealed MBE-Grown AlN on h-BN. Materials.

[40] Chen M., Zhang Q., Fang C., Shen Z., Lu Y., Liu T., Tan S., Zhang J. (2023). Influence of Sapphire Substrate with Miscut Angles on Hexagonal Boron Nitride Films Grown by Halide Vapor Phase Epitaxy. CrystEngComm.

[41] Kobayashi Y., Akasaka T. (2008). Hexagonal BN Epitaxial Growth on (0001) Sapphire Substrate by MOVPE. J. Cryst. Growth.

[42] Lin M., Huang Y., Ran C., Dong G., Zhao Y. (2024). Study on Sintering Properties of Aluminum Oxide Nano-powder for Electronics Packaging. J. Phys. Conf. Ser..

[43] Sachdev H., Haubner R., Lux B., Nöth H. (1997). Investigation of the c-BN/h-BN Phase Transformation at Normal Pressure. Diam. Relat. Mater..

[44] Hotta M., Goto T. (2008). Densification and Microstructure of Al_2_O_3_-cBN Composites Prepared by Spark Plasma Sintering. J. Ceram. Soc. Jpn..

[45] Wolfrum A.-K., Matthey B., Michaelis A., Herrmann M. (2018). On the Stability of c-BN-Reinforcing Particles in Ceramic Matrix Materials. Materials.

[46] Machado Filho M.A., Farmer W., Hsiao C.-L., dos Santos R.B., Hultman L., Birch J., Ankit K., Gueorguiev G.K. (2024). Density Functional Theory-Fed Phase Field Model for Semiconductor Nanostructures: The Case of Self-Induced Core-Shell InAlN Nanorods. Cryst. Growth Des..

[47] Kakanakova-Georgieva A., Gueorguiev G.K., Yakimova R., Janzén E. (2004). Effect of Impurity Incorporation on Crystallization in AlN Sublimation Epitaxy. J. Appl. Phys..

